# A hybrid phenomenological finite element model of transcatheter aortic valve leaflet behaviour under non-circular annular deployment: in vitro validation and biomechanical insights

**DOI:** 10.1007/s10237-026-02112-3

**Published:** 2026-07-21

**Authors:** David Agudo, Pablo Comesaña, Sofía Suárez, Irea Lopez-Garcia, Abraham Segade, Enrique Casarejos, Cesar Veiga, Laura Busto, Victor Alfonso Jimenez-Díaz, Maximilian Kütting, Andres Iñiguez

**Affiliations:** 1 CINTECX, Department of Mechanical Engineering, Universidade de Vigo, Campus As Lagoas, Marcosende, 36310 Vigo, Galicia Spain; 2 Design and Numerical Simulation Research Group, Galicia Sur Health Research Institute, Vigo, Galicia Spain; 3 Nsilica Simulation Technologies, Nigrán, Galicia Spain; 4 Cardiovascular Research Group, Galicia Sur Health Research Institute, Vigo, Galicia Spain; 5 Cardiovascular Research Unit, Hospital Alvaro Cunqueiro, University Hospital of Vigo, Vigo, Galicia Spain; 6 New Valve Technology GmbH, Biosensors International, Hechingen, Baden-Wurtemberg Germany

**Keywords:** Transcatheter aortic valve, Finite element analysis, Pericardial tissue mechanics, Leaflet coaptation, Aortic annulus ellipticity, In vitro validation

## Abstract

Transcatheter aortic valves (TAVs) typically operate on non-circular rings, but the impact of annular ellipticity on valve mechanics remains insufficiently quantified. In this study, we present a finite element (FE) framework of a 27 mm Allegra TAV that reproduces the complete model, including stent and pericardial skirt and leaflets. The leaflets were represented using a general shell formulation that decouples the in-plane and bending responses, leading to a hybrid shell–membrane finite element model. A linear elastic constitutive law was calibrated through inverse FE analysis based on cantilever bending experiments performed on bovine pericardium. The model was validated against in vitro pulse duplicator tests for circular and highly elliptical geometries, reproducing distinctive features such as full systolic opening and the asymmetric ‘pinwheel’ pattern during diastolic closure. Once validated, the model is used to investigate the impact of annular ellipticity across six annular aortic geometries. Each geometry was evaluated in two limiting orientations of the ellipse’s major axis (0$^\circ$ and 90$^\circ$) to analyse the model response in terms of valve coaptation. These results identify annular geometry and alignment as major factors in valve leaflet coaptation asymmetry and this asymmetry was found to correlate with increased stress concentration, which could compromise long-term valve function.

## Introduction

Nowadays, numerical simulation models are widely used for the analysis of devices and situations that are difficult to reproduce in laboratory analysis. This is the case for medical prostheses such as transcatheter aortic valves (TAV), where the elaboration of Finite Element (FE) models provides us with the possibility of evaluating their function prior to the intervention.

In the manufacturing of TAVs, one of the most commonly used materials is bovine or porcine pericardium (Soares et al. 2016). The pericardium is a thin membrane that covers the heart of mammals and exhibits excellent mechanical properties. It is an anisotropic material due to the collagen fibers embedded in its structure, which impart direction-dependent mechanical behavior. Numerous studies have demonstrated this anisotropy through uniaxial and biaxial tensile tests (Sacks et al. 1994; Sacks and Chuong 1998; Caballero et al. 2017; Murdock et al. 2018), revealing different stress–strain responses along preferred and cross-preferred fiber orientations. Different finite element (FE) studies have focused on accurately capturing these directional properties by developing complex material models that replicate the anisotropic behavior of pericardium. Some of them (Martin and Sun 2015; Travaglino et al. 2020; Suárez et al. 2022) use the Holzapfel-Gasser-Ogden (HGO) model (Holzapfel et al. 2000), which accounts for the contribution of collagen fibers within a hyperelastic framework, while other studies (Sun et al. 2005; Li and Sun 2010, 2017; Kim et al. 2008) used Fung-type model (Fung 1993) which provides a nonlinear anisotropic formulation, capturing the pericardium’s stiffening response under high deformations. These material models allow for a detailed representation of the mechanical behavior of pericardial tissue but their high level of complexity significantly increases the computational cost and often introduces convergence difficulties in finite element simulations.

Another aspect to consider is the manufacturing process. The fiber orientation in pericardium is not homogeneous, which poses a significant limitation for manufacturing applications. Statistical analyses of pericardium samples (Stieglmeier et al. 2021) have revealed regions with different preferred fiber orientations, but the dimensions of these regions are generally too small to ensure the fabrication of valve leaflets with a consistent and defined fiber alignment. As a result, there is no evidence that bioprosthetic heart valve (BHV) leaflets are manufactured based on any prescribed fiber orientation (Braile et al. 1998; Stieglmeier et al. 2021). In addition to this spatial variability, the intrinsic heterogeneity of the biological tissue introduces further differences in mechanical response–even among samples with similar fiber orientations–as shown by several series of uniaxial tests (Whelan et al. 2019; Jin et al. 2021). For these reasons, and in line with the uncertainty regarding leaflet fiber orientation in manufactured TAVs, we assume an isotropic elastic behavior for the leaflet material in the present FE model.

For the mechanical characterization of pericardium, planar mechanical behavior has been thoroughly explored (Sacks and Chuong 1998; Paez et al. 2003; Gauvin et al. 2013; Aguiari et al. 2016; Caballero et al. 2017), typically using uniaxial or biaxial tensile tests. However, the most relevant functional behavior of the leaflets in bioprosthetic valves is associated with their bending properties (Sacks and Yoganathan 1484). Despite its importance, the bending behavior of pericardial tissue has received little attention. Some attempts have been made to assess flexural behavior using three-point bending configurations (Engelmayr et al. 2003; Mirnajafi et al. 2005), but due to the low resistance of pericardial tissue to bending, these experiments are often challenging to perform reliably. An alternative approach proposed by Murdock et al. (2018) involves beam bending tests to evaluate the tissue’s flexural response, which provides a more robust framework. In this work, the experimental data reported by Murdock et al. (2018) are used as a reference for the isotropic model calibration used in our computational model.

Given these limitations and the high computational cost of fully nonlinear anisotropic material formulations, many finite element (FE) studies analyzing the performance of complete valves comprising the stent, skirt, and leaflets–opt for simplified material definitions. Linear elastic models are commonly used to represent the mechanical behavior of the pericardium in global simulations (Auricchio et al. 2013; Morganti et al. 2016; Amindari et al. 2017; Pasta et al. 2021; Pandya et al. 2023), even in studies focused on leaflet dynamics where the full opening and closing cycle is reproduced (Grossi et al. 2024). These simplifications allow for realistic and computationally efficient analyses while still capturing the essential features of valve kinematics.

Regarding the finite element formulation used for the leaflets, solid, shell, and membrane elements can all be considered. In the present problem, however, the choice of formulation was mainly driven by the need to reproduce the macroscopic mechanical behaviour of thin pericardial leaflets while maintaining a computationally efficient model suitable for full valve simulations with contact. The use of solid elements is generally not practical for this application because of the very small thickness of the tissue and the associated computational cost. Accurate bending representation would require several elements through the thickness, substantially increasing the total number of elements and degrees of freedom. Membrane elements provide an efficient formulation for reproducing in-plane tensile behaviour, but they do not include bending stiffness, leading to unrealistic folding behaviour and numerical difficulties during leaflet coaptation and closure. The shell formulation is employed when one dimension of the geometrical problem, typically the thickness, is significantly smaller than the other dimensions. In such cases, the stress component normal to the thickness is assumed negligible (plane-stress condition), as commonly adopted for thin-walled structures. The thickness strain, however, is not constrained and is determined by the linear elastic constitutive response. Using the shell formulation enables a much more computationally efficient model without compromising the accuracy of the stress state representation, making it ideal for in-plane stress or bending-dominated problems. This approach is particularly suitable for the pericardium, making shell elements the preferred formulation for modeling the leaflets (Sun et al. 2005; Li and Sun 2010, 2017; Travaglino et al. 2020). In most cases, quadrilateral large-strain shell elements are used. These shell elements assume that displacement varies linearly through the thickness, and the stress distribution is derived from this kinematic assumption. While this formulation is well established for thin-walled structures, its direct application to biological tissues such as pericardium may introduce an effective bending stiffness associated with classical plate theory that does not necessarily reflect the macroscopic mechanical response of the leaflet (Stella and Sacks 2007; Kim et al. 2007). To address these limitations, advanced shell elements have been developed that independently characterize in-plane and bending responses. These can be implemented using stress-resultant-based shell models (Simo and Fox 1989), as employed in the models of Kim et al. (Kim et al. 2007, 2008), or through a general shell section definition within the Abaqus FE framework, which is the approach adopted in this study, where a hybrid shell–membrane finite element model is developed.

With regard to the stent, it forms the main structural body of the TAV and consists of a mesh-like cylindrical frame that provides support to the prosthetic leaflets and allows the device to anchor within the native aortic root. It has gold markers that allow the surgeon to know the position and orientation of the TAV during its deployment in the aorta. The material used to manufacture the stent is Nitinol, a superelastic material. In studies that reproduce the full transcatheter delivery and deployment process, the nitinol is modeleded as a superelastic material to capture the phase-transformation response characteristic of nitinol (Morganti et al. 2016). In the present work, the geometric constraint applied to the stent remains closer to physiologically relevant loading ranges. For such deformation levels, Hopf et al. (Hopf et al. 2017) provided evidence that the simplification to a linear elastic constitutive law can be justified at the global scale. Therefore, in this study the stent is modeled using a linear elastic material law.

This study aims to provide a reliable and computationally efficient model to capture the motion of the valve leaflets. In contrast to many existing analyses, which primarily focus on the closed configuration of the valve (Abbasi and Azadani 2015; Li and Sun 2017) or on device deployment during the implantation procedure (Morganti et al. 2014; Zhang et al. 2025), the present work investigates both the opening and closing configurations of the valve and analyzes the asymmetric leaflet motion induced by deployment within elliptical aortic root geometries.

The remainder of this article is structured as follows. First, we present the development of a hybrid finite element model designed specifically to accurately capture the flexural behavior of pericardial tissue, as observed in specific bending tests. This section includes the modeling strategy, element formulation, and calibration process based on experimental data. Next, we describe the construction of a complete virtual model of a transcatheter aortic valve (TAV), incorporating the stent, skirt, and leaflets. In the next step, we present the results, including a comparison of leaflet dynamics with in vitro experiments and an analysis of the differential behavior of the leaflets associated with various aortic root anatomies. Finally, the article concludes with a discussion of the main findings and their implications.

## Metodology

### Characterization of the pericardial tissue

The construction of the virtual valve model began with the definition of the computational model for the pericardial tissue. As noted previously, while many studies adopted anisotropic formulations to account for the fibrous structure of the pericardium, this approach was not followed here due to the difficulty in ensuring consistent fiber alignment during valve leaflet manufacturing, as found in dedicated studies. The objective was to develop a hybrid shell–membrane finite element model capable of accurately reproducing the bending behavior of the tissue while maintaining good numerical convergence and computational efficiency.

#### Element formulation

The goal of the element formulation was to represent both the in-plane and out-of-plane mechanical response of the pericardial tissue. This was necessary to obtain a material characterization capable of reproducing both tensile and flexural test conditions, while maintaining a computationally efficient formulation for the simulation of valve leaflet mechanics.

In this work, the general shell section was defined using a hybrid shell–membrane finite element approach. The formulation was based on generalized shell stress resultants, as used in the Abaqus general shell section formulation.

The vector containing the generalized shell stress resultants is defined as in (1), where the first three components ($N_x$, $N_y$, and $N_{xy}$) correspond to the membrane forces per unit length associated with the in-plane response of the shell, whereas the last three components ($M_x$, $M_y$, and $M_{xy}$) correspond to the bending moments per unit length associated with the out-of-plane response.

1 $$\begin{aligned} \textbf{N}= \begin{bmatrix} N_{x}\\ N_{y}\\ N_{xy}\\ M_{x}\\ M_{y}\\ M_{xy} \end{bmatrix}. \end{aligned}$$

The generalized shell section stiffness matrix $\textbf{D}$ relates these generalized shell stress resultants $\textbf{N}$ to the generalized shell strain vector $\textbf{E}$. The generalized strain vector contains the membrane strain components and the curvature-related bending strain components, and can be written as

2 $$\begin{aligned} \textbf{E}= \begin{bmatrix} \varepsilon _x\\ \varepsilon _y\\ \gamma _{xy}\\ \kappa _x\\ \kappa _y\\ \kappa _{xy} \end{bmatrix}. \end{aligned}$$

The constitutive relation for the general shell section was then written in matrix–vector form as

3 $$\begin{aligned} \textbf{N}=\textbf{D}\textbf{E}-\textbf{N}^{th}. \end{aligned}$$

where $\textbf{N}$ is the vector of generalized shell stress resultants, $\textbf{D}$ is the shell section stiffness matrix, $\textbf{E}$ is the generalized shell strain vector, and $\textbf{N}^{th}$ is the vector of thermal generalized stress resultants.

For the present hybrid shell–membrane formulation, the stiffness matrix $\textbf{D}$ took the form shown in (4):

4 $$\begin{aligned} \textbf{D}= \begin{bmatrix} \frac{tE}{1-\nu ^2}& \frac{t\nu E}{1-\nu ^2}& 0& 0& 0& 0\\ \frac{t\nu E}{1-\nu ^2}& \frac{tE}{1-\nu ^2}& 0& 0& 0& 0\\ 0& 0& \frac{tE(1-\nu )}{2(1-\nu ^2)}& 0& 0& 0\\ 0& 0& 0& \eta \frac{Et^3}{12(1-\nu ^2)}& \eta \frac{\nu Et^3}{12(1-\nu ^2)}& 0\\ 0& 0& 0& \eta \frac{\nu Et^3}{12(1-\nu ^2)}& \eta \frac{Et^3}{12(1-\nu ^2)}& 0\\ 0& 0& 0& 0& 0& \eta \frac{Et^3}{12(1-\nu ^2)}\left( \frac{1-\nu }{2}\right) \end{bmatrix}. \end{aligned}$$

Since the stiffness matrix is a 6×6 symmetric matrix, only 21 independent terms need to be specified: the 6 diagonal terms and the 15 off-diagonal terms. In (4), *E* denotes the Young’s modulus of the material, ν is the Poisson ratio, and *t* is the shell thickness. The thickness was measured prior to mechanical testing, whereas the Young’s modulus was obtained by linearizing the high-stiffness region of the tensile stress–strain response at large strains. The parameter η is a flexural correction factor introduced to adjust the bending stiffness of the shell section (Cook et al. 1989) Although this parameter does not affect the in-plane membrane stiffness, it plays a central role in controlling the out-of-plane bending response of the shell element.

Because the general shell section formulation provides generalized section resultants rather than directly outputting local three-dimensional stresses, a post-processing procedure was required to obtain an equivalent scalar stress measure. A Python script was therefore used to extract the membrane force resultants and bending moment resultants from the shell section output and convert them into equivalent local stress-like quantities.

The membrane contribution was obtained from the section force resultants as

5 $$\begin{aligned} \textbf{S}^{SF}=\frac{\textbf{SF}}{t}, \end{aligned}$$

where $\textbf{SF}=[N_x,\,N_y,\,N_{xy}]^T$ is the vector of membrane force resultants and *t* is the shell thickness.

The bending contribution was obtained from the section moment resultants as

6 $$\begin{aligned} \textbf{S}^{SM}=\frac{6\textbf{SM}}{t^2}, \end{aligned}$$

where $\textbf{SM}=[M_x,\,M_y,\,M_{xy}]^T$ is the vector of bending moment resultants. This expression corresponds to the stress contribution associated with a linear through-thickness bending stress distribution evaluated at the outer surface of the shell.

The equivalent von Mises contribution associated with the membrane force resultants was then calculated as

7 $$\begin{aligned} \textrm{VM}^{SF} = \sqrt{ \left( S_1^{SF}\right) ^2 + \left( S_2^{SF}\right) ^2 + 3\left( S_3^{SF}\right) ^2 - S_1^{SF} S_2^{SF} }. \end{aligned}$$

Similarly, the equivalent von Mises contribution associated with the bending moment resultants was calculated as

8 $$\begin{aligned} \textrm{VM}^{SM} = \sqrt{ \left( S_1^{SM}\right) ^2 + \left( S_2^{SM}\right) ^2 + 3\left( S_3^{SM}\right) ^2 - S_1^{SM} S_2^{SM} }. \end{aligned}$$

Based on this data, the Von Mises stress value was calculated as

9 $$\begin{aligned} \textrm{VM}^{TOT} = \textrm{VM}^{SF} + \textrm{VM}^{SM}. \end{aligned}$$

#### Calibration of pericardium model: benchmark FE simulation

To calibrate the parameters of the pericardium material model described above, experimental data from a benchmark beam-bending test were taken from the literature. In particular, the cantilever beam-bending experiment reported by Murdock et al. (2018) was used as the reference experimental dataset for evaluating the flexural response of pericardial tissue. The experimental procedure is described in detail in that study; therefore, only the main features relevant to the present calibration are briefly summarized here.As described by Murdock et al. (2018), pericardium samples measured $12 \times 5.75\,\textrm{mm}$ with a thickness of $0.32\,\textrm{mm}$, consistent with the dimensions previously reported by Caballero et al. (2017). In that setup, one end of the specimen was clamped while the other remained free, and bending was induced by attaching a needle to the free end of the tissue through a suture thread. Two needle masses, 56 mg and 125 mg, were considered for each sample. The displacement data reported in Murdock et al. (2018) were then used for the calibration and validation of the finite element model of the pericardial tissue.

**Fig. 1:**
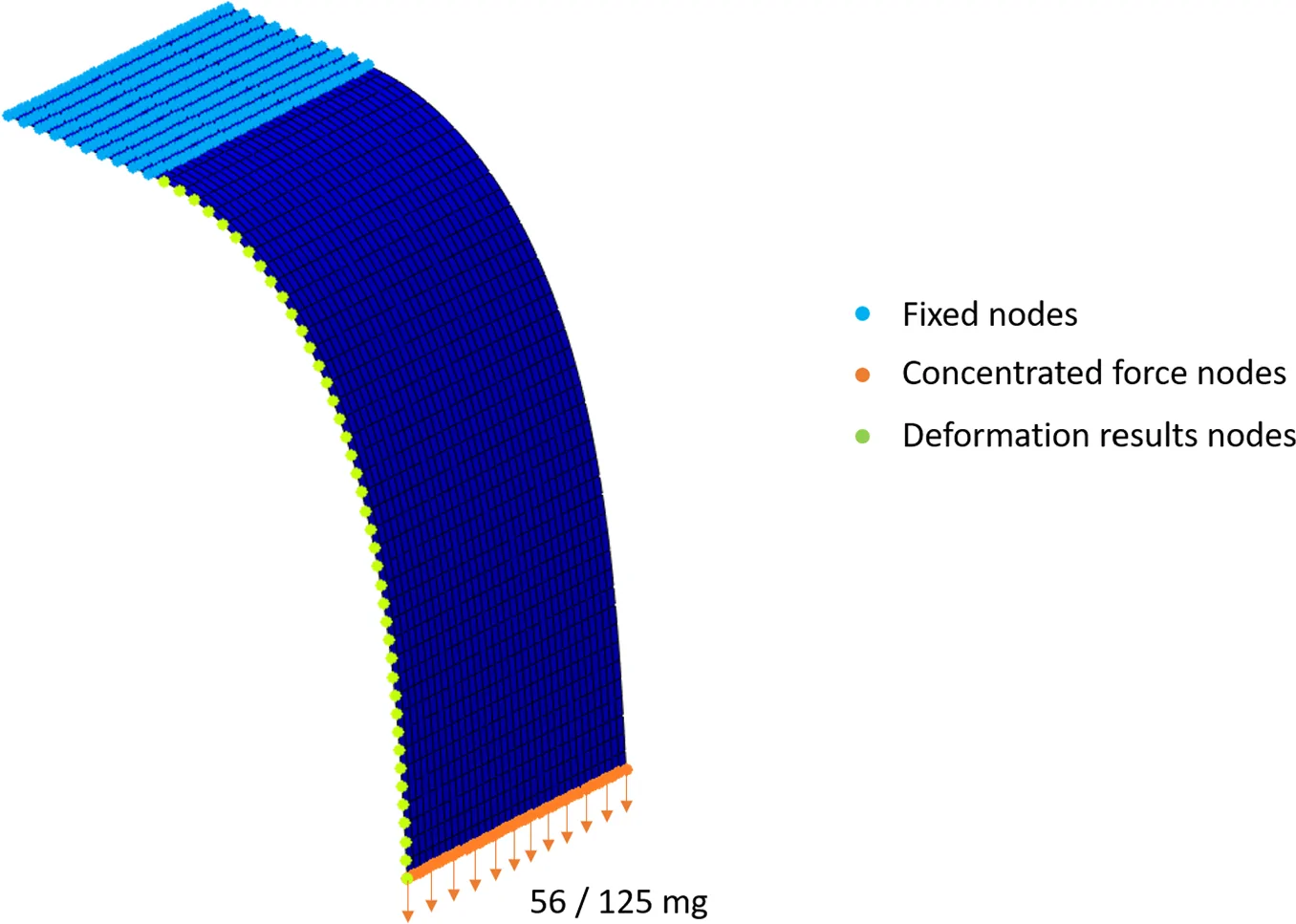
Benchmark finite element model of a cantilever beam sample of bovine pericardium under boundary conditions mimicking the real experimental test conditions

The benchmark Finite Element (FE) model was developed using 1,683 shell continuum elements (Abaqus S4), with 51 elements along the length and 33 elements along the width. The cantilever bending experiment was simulated by fixing a section of 10×34 nodes at one end, leaving a 12 mm portion of the length free (Fig. 1), simulating the conditions of the experimental setup. A concentrated force was applied to the free end to mimic the experimental protocol, with two force conditions simulated: 56 mg and 125 mg. Additionally, the weight of the sample and its buoyancy in the aqueous medium were modeled as volumetric loads. The tissue deformation caused by each weight was recorded by tracking the position of the nodes along the tissue length and compared with the actual deformation observed in the experimental tests.

Four material parameters are needed to determine the stiffness matrix. A leaflet thickness of 0.32 mm was assumed (Caballero et al. 2017), together with a Poisson’s ratio of 0.45, consistent with values reported in the literature (Luraghi et al. 2021; Grossi et al. 2024; Pandya et al. 2023). Therefore, the only two parameters that need to be adjusted to match the behavior of our cantilever finite element model with the experimental tests are the Young’s Modulus (*E*) and the flexural correction factor η.

#### Calibration of pericardium model: inverse finite element analysis

To identify the pericardial material parameters that best reproduce the experimental flexural response, an inverse finite element analysis was carried out. A Latin Hypercube Sampling (LHS) approach was used to generate 50 quasi-random pairs of values within a predefined parameter domain. Based on values reported for bovine pericardium in the literature, the Young’s Modulus was sampled in the range $E \in [4,\,100]$ MPa. The flexural correction factor is dimensionless and sampled in the range $\eta \in [0,\,1]$. Each parameter pair was used to simulate the beam-bending test for both load cases (56 mg and 125 mg), and the resulting deflections were compared to the experimental values. The Root Mean Square Error (RMSE) was calculated from the nodal differences between the simulated and experimentally measured deflections.

Following this parametric analysis, a sequential neural network model was trained to approximate the RMSE response surface. The model took *E* and η as inputs and consisted of 15 hidden layers, each containing 50 neurons with Rectified Linear Unit (ReLU) activation. The output layer included a single neuron for regression, with no activation function. The model was compiled using the Adam optimizer, and the RMSE was used as the loss function.

**Fig. 2:**
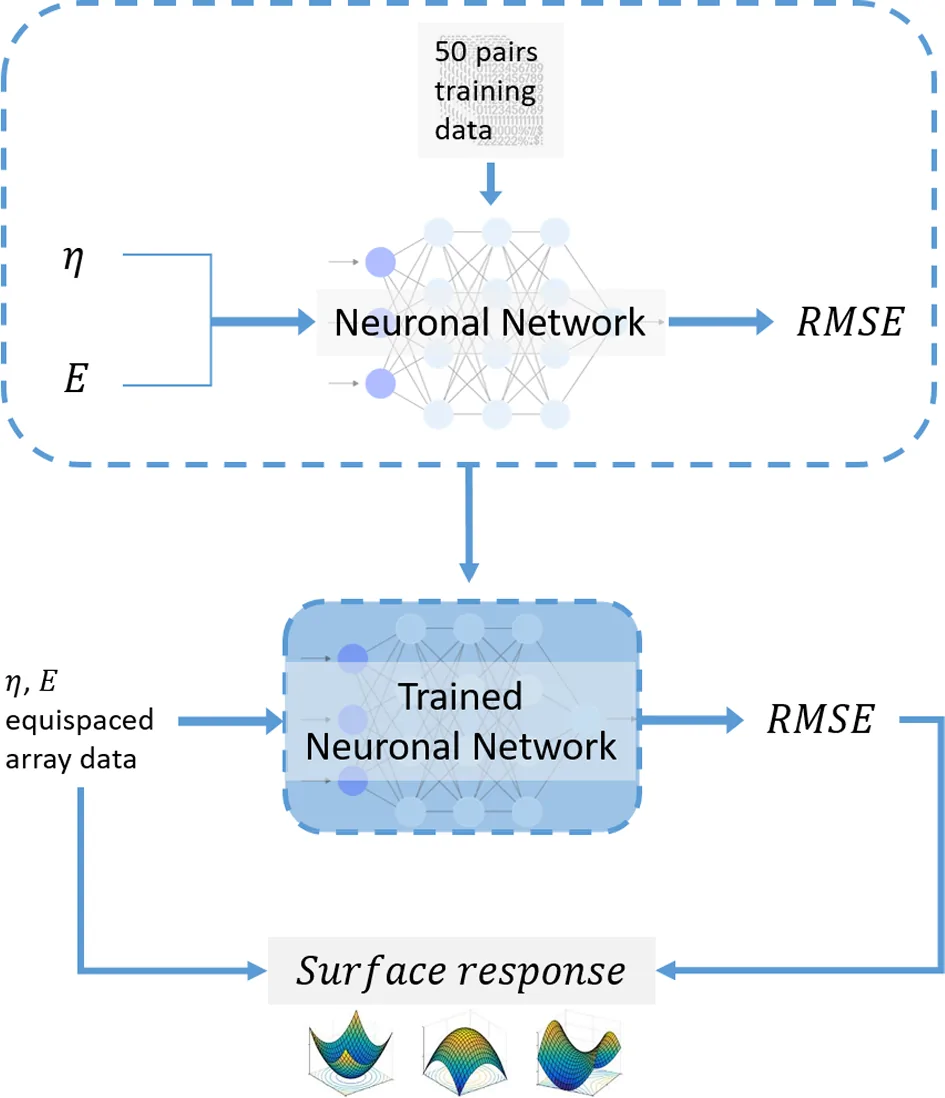
Scheme of application of a neural network to obtain the response surface

The model was trained with normalized data for η and Young’s Modulus over 200 epochs, with the output being the mean RMSE between the two loading tests. The result was a trained neural network model used to evaluate the RMSE for an array of η and Young’s Modulus values, consisting of 40 values of η ranging from 0 to 1 in increments of 0.025, and 38 values of Young’s Modulus ranging from 4 to 100 in increments of 2.5. The result was a response surface that shows the RMSE levels for each point in the array. Figure 2 presents a schematic representation of the application of the developed Neural Network, where η and Young’s Modulus (*E*) served as input parameters. The resulting response surface illustrated the correlation between the numerical material characterization and the experimental tests.

**Fig. 3:**
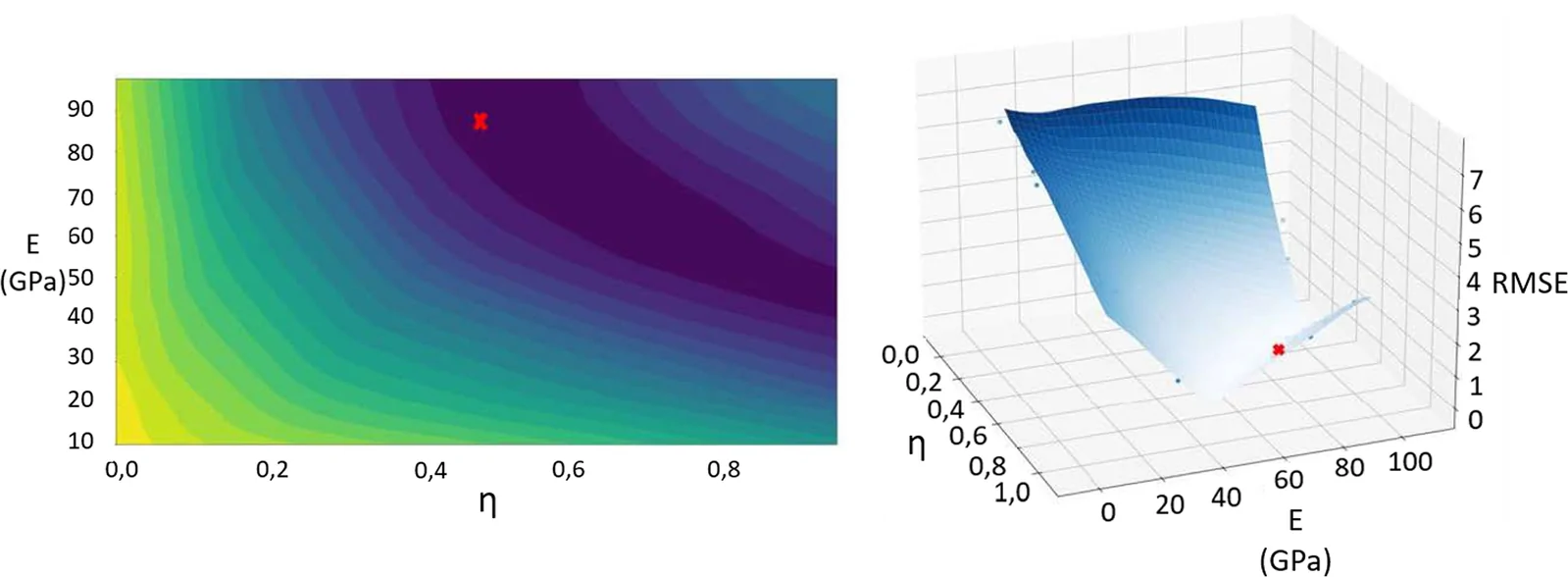
Response surface obtained from the inverse analysis. The minimum RMSE is highlighted in red. Left: 2D colour representation, where colour indicates the error magnitude. Right: 3D representation of the same response surface, where the error magnitude is represented along the vertical axis

This response surface, shown in Fig. 3, was analysed to identify the minimum RMSE and the corresponding η and *E* values. This figure shows two ways of presenting the response surface obtained, both with the lowest error point marked in red; however, whilst the image on the left uses a 2D representation with colour to indicate the error magnitude, the image on the right uses a 3D representation where the error magnitude is represented along the vertical axis.

#### Material parameters

**Fig. 4:**
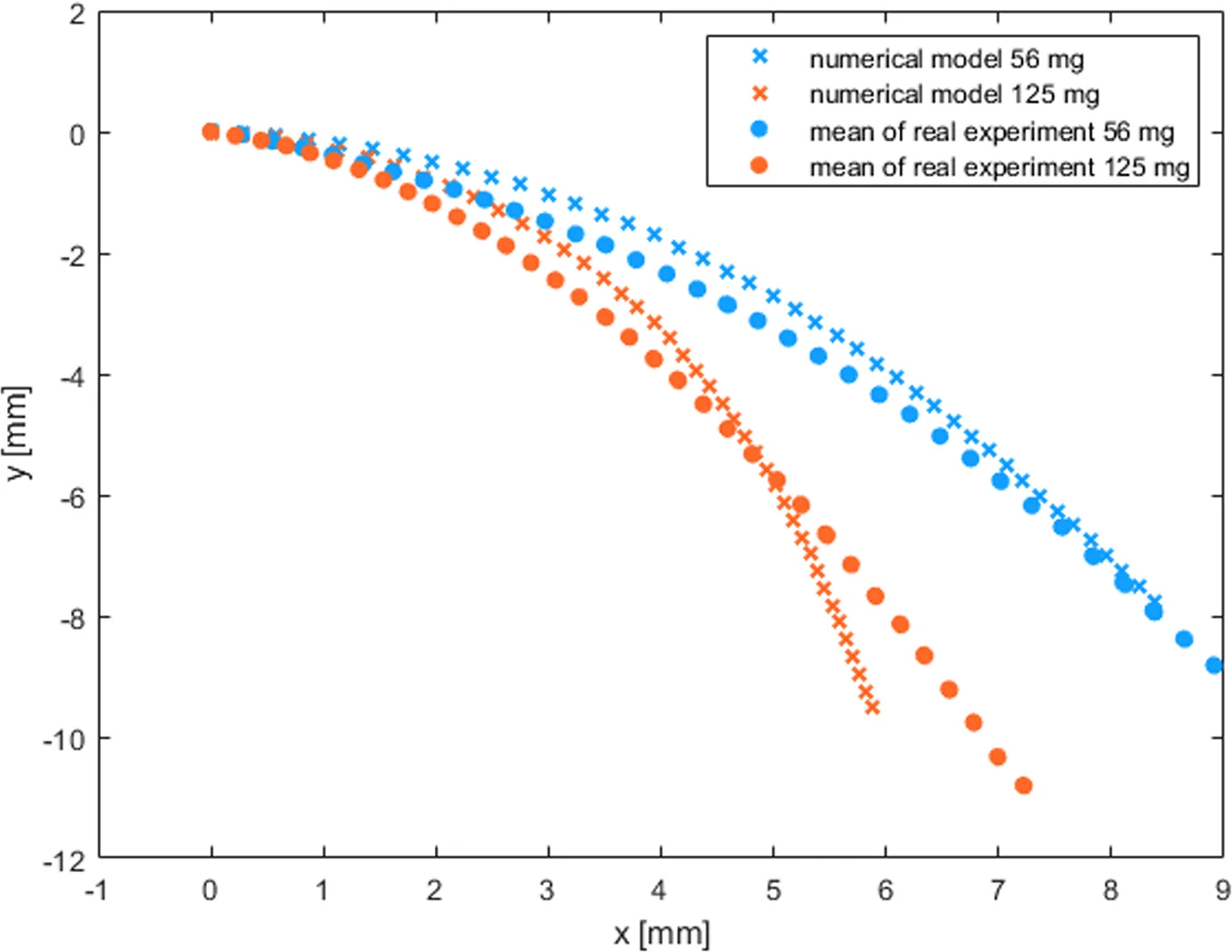
Comparison between real deformation and the deformation obtained from simulation for the cantilever bending model that best represents the behaviour of the material. The x- and y-axis scales are not identical, following the representation format used in Murdock et al. (Murdock et al. 2018), in order to facilitate comparison between results

The final values obtained from the inverse finite element analysis were a Young’s Modulus of $89\,\textrm{MPa}$, which is consistent with reported ranges for bovine pericardium literature (Sanchez-Arevalo et al. 2010), and η=0.525. The Root Mean Square Error (RMSE) for each weighted model is 0.4300 mm for the 56 mg model and 0.7526 mm for the 125 mg model, resulting in a mean error of 0.5913 mm between the two models. Figure 4 shows a comparison between real and simulated deformations for both models.

The fitting obtained for the 56 mg case is slightly better than that achieved for the 125 mg case. Nevertheless, in both cases, the numerical response remains within the variability range reported by Murdock et al. (Murdock et al. 2018) for bovine pericardium specimens under comparable bending conditions. Therefore, the calibration was considered adequate to obtain a representative flexural response of the pericardial tissue.

### Virtual model of 3D TAV device

The Finite Element Model of a TAV must accurately replicate the various characteristics of the actual prosthesis, from its geometry to the boundary conditions under which it operates. In this study, a complete TAV model has been simulated. The reference used is a 27 mm ALLEGRA Transcatheter Heart Valve (NVT GmbH, Biosensors International, Hechingen, Germany). The TAV itself comprises two main components: the metallic stent and the tissue elements, which include the leaflets and the skirt, where the leaflets are sutured, and the stent is anchored. Additionally, it will be necessary to define the external geometry, the aortic wall, for which a simplified cylindrical model will be used, based on measurements taken from patients and in vitro tests.

To simulate the behavior of the prosthetic valve, a finite element model was constructed using an implicit dynamic integration method, focused on reproducing the mechanics of the valve rather than its fluid–structure interaction. All finite element simulations were performed using Abaqus/Standard 2022 (Dassault Systèmes Simulia Corp., Providence, RI, USA. In implicit analyses, an initial time increment is prescribed for each step, and Abaqus automatically adjusts the increment size throughout the simulation based on convergence behavior. Large-deformation effects were included by activating the NLGEOM option, which enabled a geometrically nonlinear formulation: the equilibrium equations were written with respect to the current (deformed) configuration, and kinematic nonlinearities due to large displacements and rotations were accounted for.

The numerical model was contrasted with experimental in vitro measurements obtained for two specific configurations, allowing the simulated valve response to be compared with the behaviour observed in laboratory in vitro tests. The following subsections describe the modeling and meshing techniques employed to achieve precise geometries and well-structured, homogeneous meshes (Tables 1, 2 and 3).

#### Stent modelling

The simulation model was created from a stereolithography file (STL) provided by the manufacturer. From this file, a solid mesh was developed using regular hexahedrons in its construction. The mesh consisted of five layers across the thickness, requiring a total of 201,260 C3D8R-type hexahedral elements, achieving the quality results shown in Table 4, which is in Appendix A.

For the characterization of Nitinol, the results of three experimental in vitro radial tests provided by the manufacturer were used, which yielded force-diameter curves. The characterization focuses on the elastic range of the superelastic memory alloy curve as Hopf et al.(Hopf et al. 2017). The linear elastic parameters of the material were derived from the manufacturer curves, using the section of the curve that reflects the linear elastic behaviour of austenite, and yielding an average Young’s modulus of 50.74 GPa. A typical Poisson’s ratio of 0.33 was assumed based on values reported in the literature (Meng et al. 2024; Castravete et al. 2020).

**Fig. 5:**
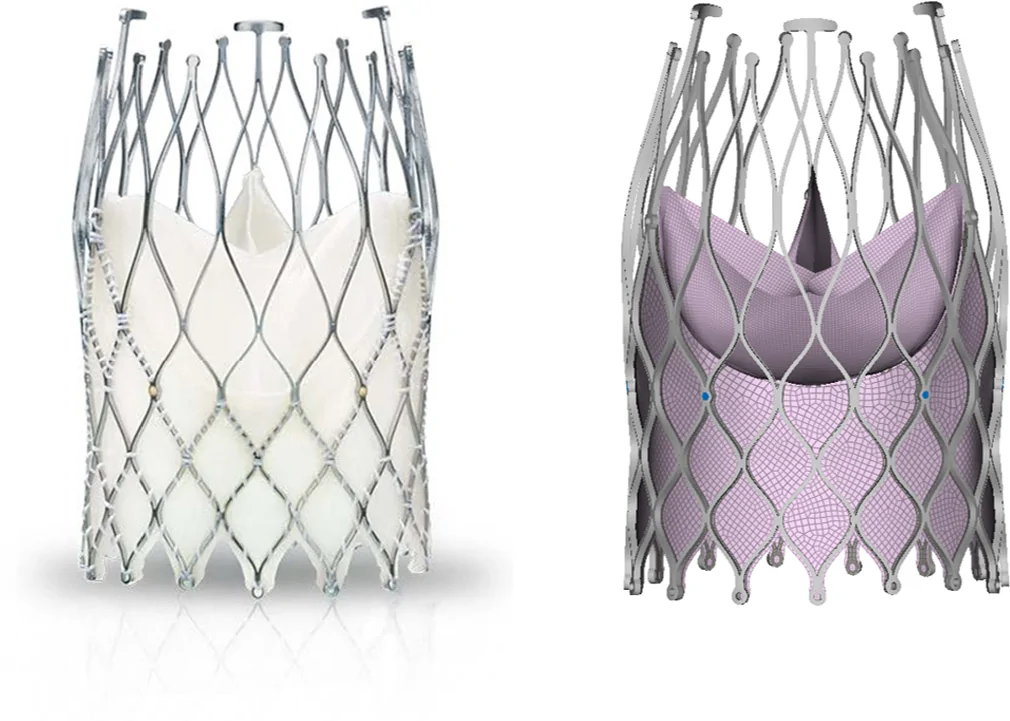
Comparison between the real ALLEGRA transcatheter aortic valve and the corresponding finite element model developed in this study. Two of the valve gold markers are visible in the real device picture with their characteristic gold colour, whereas the corresponding markers are represented in blue in the FEM model

The final mesh is shown in Fig. 5, where you can see how similar the models are, and which includes details such as the Gold Points, shown in their characteristic gold colour in the real model, whilst in the simulation model they are shown in blue.

#### Tissue components: leaflets and skirt modelling

**Fig. 6:**
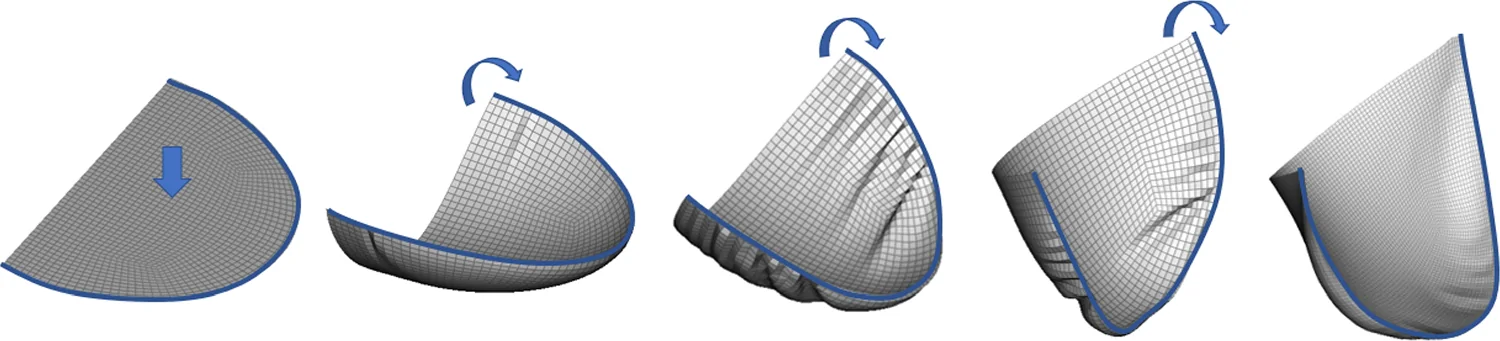
Transformation of the leaflet geometry from its initial configuration: flat pericardial tissue after cutting of the leaflet pattern, displacement of the elliptical border from the planar configuration to the final three-dimensional geometry connected to the skirt, and final mesh smoothing and refinement

The tissue components of the valve are the leaflets and the skirt, which are made from bovine pericardium. The leaflets are responsible for replicating the movements and function of the native valve. It is a thin tissue attached through stitching points, making these points crucial for ensuring the valve’s proper behavior. The pericardium of the leaflets will be defined with the previous mechanical properties; Young Modulus of 89*MPa*, Poisson ratio of 0.45, thickness of 0.32*mm* and η=0.525 (Sanchez-Arevalo et al. 2010; Caballero et al. 2017). The geometry of the leaflets for the virtual model can be obtained through 3D scanning (Sun et al. 2005), while recent publications derive it through numerical simulation, mimicking the sewing process (Li and Sun 2017; Travaglino et al. 2020). The second method was selected, in which the starting point was a flat pericardium tissue, with thickness, shape, and dimensions provided by the manufacturer. The geometry was meshed with 1,500 elements, gravity was applied to account for the weight of the pericardium, and a displacement (Fig. 6) was imposed on the elliptical border of the leaflet, shifting it from the flat tissue position to the three-dimensional final geometry that connects it with the skirt. The accuracy of the resulting leaflet geometry was assessed by comparing the initial configuration against the target final surface, revealing a surface difference of less than 3% of the sum of the element areas, thus confirming the accuracy of the shape reconstruction process.

The final geometry is then refined by increasing the mesh density to improve the accuracy of the results, from the initial 1500 elements used for geometry definition to 6,200 S4 shell elements per leaflet in the final evaluation model. It is then converted from a Shell Section to a General Shell Section. The mesh quality details are shown in Table 5, which is in Appendix A.

The skirt is a piece of tissue that connects the leaflets to the stent, preventing the need to stitch the leaflets directly to the stent. While the skirt does not serve any additional structural function, it creates a closed space for blood flow, mitigating the negative impact of the stent’s metallic structure. The skirt mesh was constrained by the need for spatial alignment between the external stent nodes and the skirt nodes, as well as between the elliptical border nodes of the leaflets and the skirt nodes. These two constraints, along with the skirt’s geometry, are the key factors that define the mesh.

For this reason, we have chosen to model this component in a simpler way than Leaflets. To do this, a linear elastic material (Young Modulus of 89 MPa and a Poisson´s ratio of 0.45) (Sanchez-Arevalo et al. 2010; Caballero et al. 2017) was used to characterize the material, and its geometry was modeled using a mesh of approximately 8,860 elements, composed of triangles (S3) and quadrilaterals (S4R). Table 6, which is in Appendix A, summarized the quality parameters of the skirt mesh.

#### Boundary conditions, contacts and loads

Aortic dimensions vary considerably between individuals, influenced by factors such as sex, age, and race (Patel et al. 2022). Studies (Lehmkuhl et al. 2013; Maeno et al. 2017; Abdel-Wahab et al. 2014) establish that different aortic phenotypes are critical for the proper function of the final device. In this study, the valve behavior was analyzed when the stent was constrained within idealized cylindrical aortic root geometries with constant elliptical cross-sections and varying degrees of ellipticity. To characterize annular geometry, the Aortic Annulus Ratio (AAR)–defined as the ratio between the maximum and minimum annular diameters–is clinically recognized (Kappetein et al. 2012). For the sake of model simplification and to enable a clearer evaluation of leaflet dynamics in both in vitro and FEM analyses, we have used a constant section and defined the parameter Ellipticity (*E*), equivalent to the AAR, as the ratio between the maximum diameter ($D_{\max }$) and the minimum diameter ($D_{\min }$) of the annular cross-section. Values close to E=1.0 indicate a circular geometry, E=1.1-1.2 correspond to mildly elliptical geometries, and E=1.3-1.5 represent highly elliptical configurations (always with constant cross-section). It should be noted that, due to the supra-annular design of the Allegra valve, the device would not operate under such extreme ellipticity values, in real conditions, although the inflow section may present significant ellipticity (E = 1.3–1.5), the elasticity of the stent frame partially compensates for this deformation along the mid and outflow sections (Busto et al. 105991, 2023), where the leaflets are anchored, resulting in lower effective ellipticity during valve operation.

The selected models have values of ellipticity E=1.0 (circular), 1.06 (almost circular), 1.10 (slightly elliptical), 1.20 (moderately elliptical), 1.35 (notably elliptical) and 1.50 (very elliptical) (see Table 1).

**Table 1:** Aortic models used to perform the ellipticity analysis

Statistical relation	Denomination	Ellipticity (*E*)	Shape
Baseline	Ellipse−1.0	1.0	
Min	Ellipse−1.06	1.06	
Low	Ellipse−1.10	1.10	
Mean	Ellipse−1.20	1.20	
High	Ellipse−1.35	1.35	
Max	Ellipse−1.50	1.50	

If we compare the ellipticity values used in the present study with the eccentricity measurements reported by Busto et al. (105991) obtained from 4D CT scans of real patients implanted with the same Allegra valve model, a good correspondence can be observed. As shown in Table 2, the eccentricity values reported by Busto et al., ranging from 0.35 to 0.69 at the end of diastole, approximately correspond to the ellipticity values between E=1.20 and E=1.35 considered in this work. Therefore, except for the most extreme configuration (E=1.50), the analyzed phenotypes fall within a realistic range for this valve model, while the E=1.50 case was included as an idealized extreme condition.

**Table 2:** Equivalence between the ellipticity used in this study and eccentricity, based on the criteria set out by Busto et al.

Ellipticity (*E*)	*a*	*b*	Eccentricity
1.00	26.2	26.2	0.00
1.06	26.2	27.8	0.33
1.10	26.1	28.3	0.39
1.20	25.7	29.5	0.49
1.35	24.0	31.4	0.64
1.50	22.3	33.4	0.74

In the computational model, the boundary condition was therefore defined as a rigid cylindrical wall with a constant elliptical section, onto which the stent frame is constrained. This approach allowed the influence of ellipticity on valve deformation and leaflet kinematics to be isolated while avoiding additional geometric complexities associated with patient-specific anatomies. The mesh covering these shape was composed of regular quadrilateral elements (S4R) with a uniform mesh size of 0.5 mm for the six aortic geometry models.

The applied boundary conditions included restricting axial movement at the stent’s gold markers (Fig. 5), fully constraining the ends of the arterial model, and introducing constraints to replicate the effects of the suture points between the stent and the skirt, as well as between the skirt and the leaflets. Notably, a kinematic coupling was defined at each commissure between adjacent leaflets to reproduce the actual suturing at these junctions. Contacts between elements were managed using penalty contact formulations applied to node-to-surface or surface-to-surface configurations depending on the contact to be reproduced. Contact interactions were defined according to the requirements of each interface using a frictionless formulation Scuoppo et al. (2023); Gierig et al. (2025). This assumption was considered appropriate due to the nature of the materials involved and the fact that the components operate immersed in a fluid environment.

To simulate the opening and closing motion of the leaflets, pressure loads were applied directly to the leaflet surfaces. The magnitude and temporal variation of these loads were derived from the transvalvular pressure gradient between the ventricle and the aorta reported in Shier et al. (1996). Accordingly, a pressure difference of 100 mmHg was used to drive leaflet closing, whereas a pressure difference of 5 mmHg was used to drive leaflet opening.

**Fig. 7:**
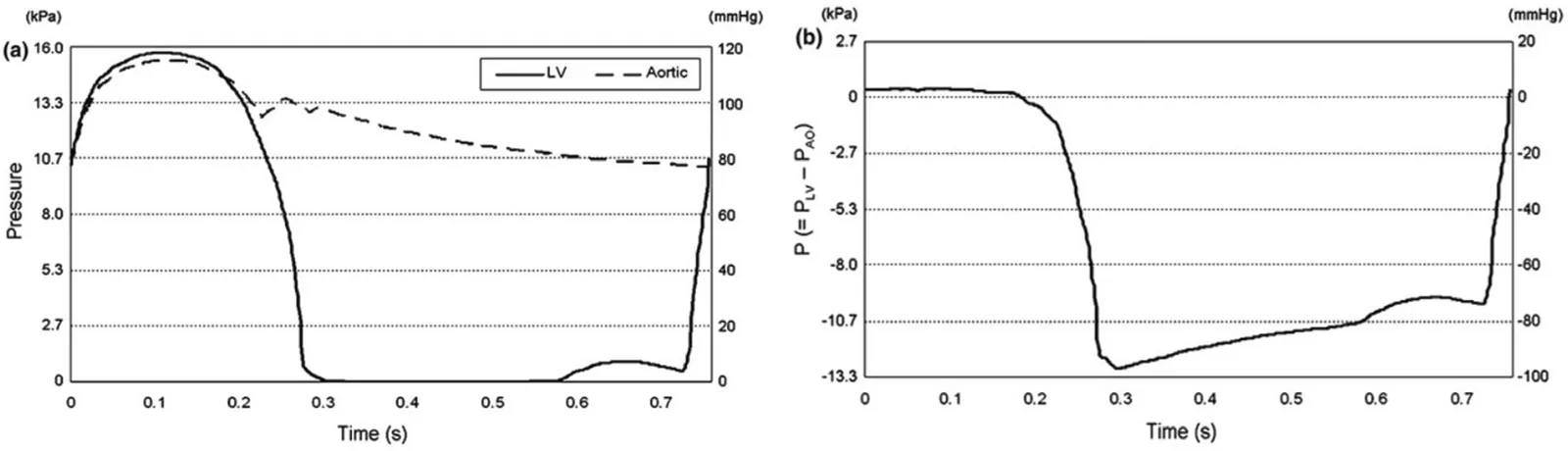
Time-varying pressure for human cardiac cycle: **a** Left ventricular and aortic pressure **b** transvalvular pressure. From Shier et al. (1996)

In this study, the finite element model was first defined using physiological boundary and loading conditions reported in the literature. The pulse duplicator experiments were then configured to reproduce the same operating conditions, which fall within the physiological range of aortic valve function. This strategy allowed the computational and experimental analyses to be performed under consistent conditions and enabled direct comparison between both approaches.

The TAV geometric model was initially larger than the target geometry that represents the simplified aortic root. Therefore, prior to simulating valve opening and closing, the valve must be compressed to fit within the elliptical rings that constrain the stent. Even in the case of a circular geometry (E=1.0), the valve diameter was reduced from its relaxed state to 26.2 mm, as this compression is always necessary for the TAV to be secured to the aortic wall.

To solve this problem, two initial steps were added to each calculation in order to achieve the geometric shape with the desired diameter and/or ellipticity in each case.

**Fig. 8:**
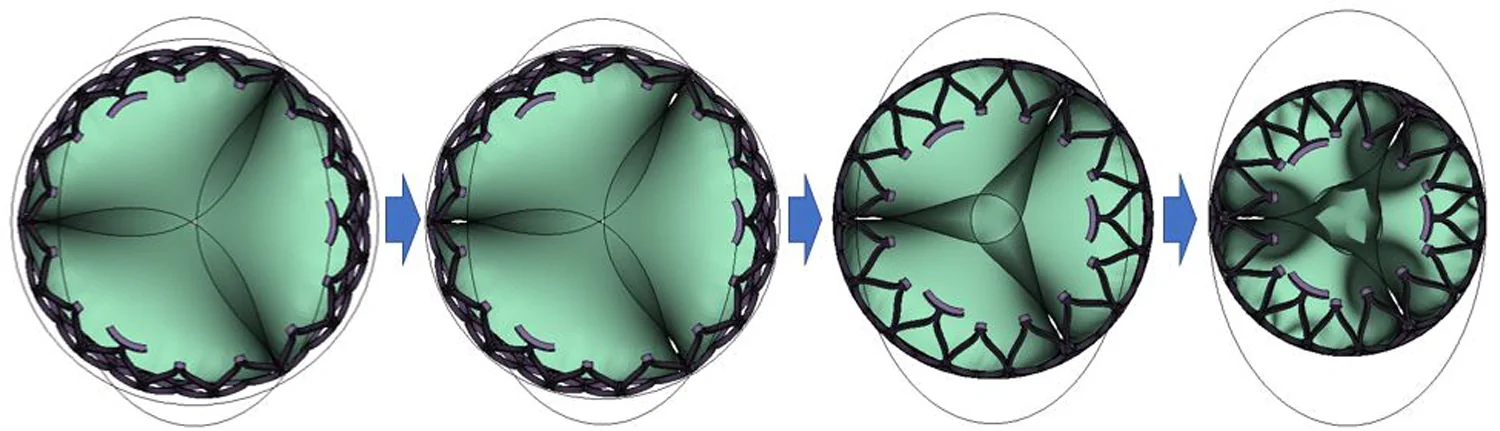
First step of the simulation: radial compression of the valve using a cylindrical surface to fully introduce it into the elliptical geometry

In the first step of the simulation, the valve must be compressed in order to fit inside the elliptical geometry. To achieve this, a cylindrical surface was defined around the valve, and its radius was progressively reduced by means of a boundary displacement. This generated a radial compression of the valve until it was fully contained within the elliptical geometry.(Fig. 8)

**Fig. 9:**
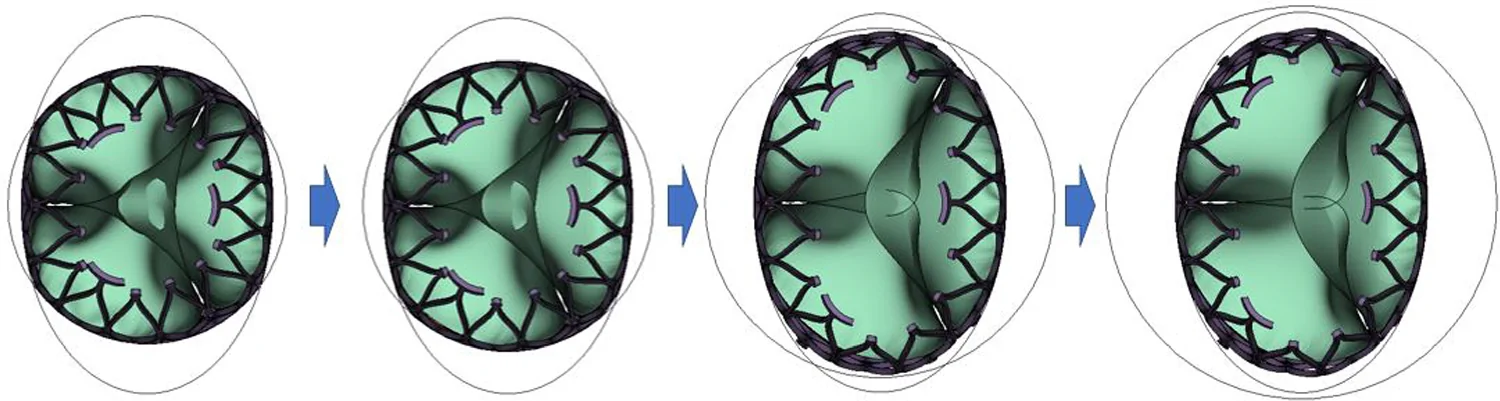
Second step of the simulation: adaptation of the valve to the eliptical geometry

In the second step of the calculation, contact between the valve and the elliptical geometry was then enabled, and the cylinder’s radius was increased back to its original value, leaving the valve confined within the limits of the elliptical shape and adapting to it according to the stresses on the stent, skirt and leaflets (Fig. 9).

Once the valve has adapted to the elliptical geometry, the valve opening and closing cycles began, as described below.

With the TAV model positioned inside the target geometry, the stent exerted pressure as it is forced to adapt to the imposed geometry. In addition, initial penetrations existed between the three leaflets because each leaflet was modeled independently according to the procedure described in Sect. 2.3.1. These inter penetrations were automatically resolved at the beginning of the valve opening and closing simulations. These simulations followed the same scheme across all three cases, consisting of four steps and a dynamic implicit scheme:

- Step 1: Start of opening, from 0 to 0.05 s. The simulation begins, establishing contacts between the leaflets to prevent collapse.
- Step 2: End of opening, from 0.05 to 0.2 s. Final contacts are established to allow a complete opening of the leaflets.
- Step 3: Start of closing, at 0.2 s. Maintains contact between the leaflets and the stent to avoid penetrations during closure.
- Step 4: End of closing, from 0.3 to 0.7 s. Final contacts between the leaflets are activated to complete the cycle.

The durations of each step are adjusted according to the natural cardiac cycle: the opening occurs within 0.2 s, while the closure extends up to 0.5 s at which point a new opening cycle begins (Fig. 7).

### Model performance for circular geometry (E = 1)

To validate the finite element model, in-vitro testing was conducted using a 27 mm Allegra transcatheter valve, which was tested in the Heart Valve Duplicator System (HDTi 6000, BDC Laboratories, USA).To carry out the in vitro test, the valve is first inserted into a ring with the desired geometry; in our case, this has a circular and elliptical geometry. This assembly is then mounted inside the test device, which is capable of replicating cardiac flow under the conditions described by Shier et al. (1996).

The first validation was performed for a valve with aortic root geometry characterized by a circular annulus (E=1), replicating the same configuration used in the experimental setup.

**Fig. 10:**
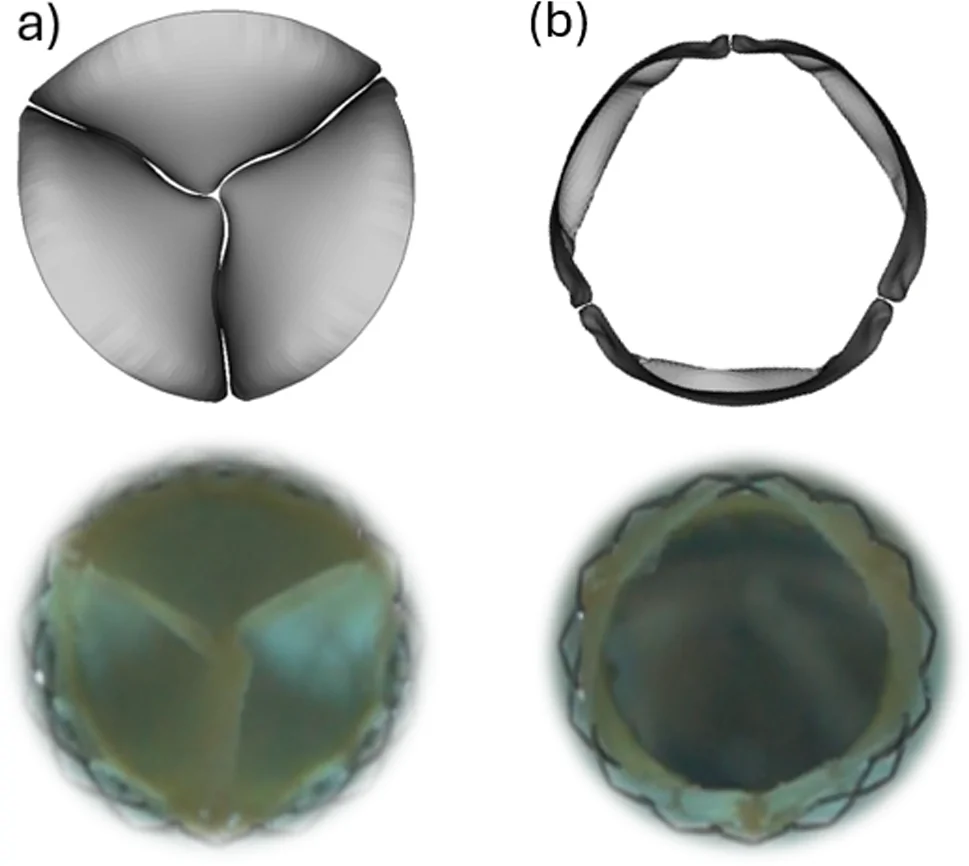
Comparison between the FE model (top row) and the in vitro test (bottom row) for E=1: **a** valve closure during diastole showing the pinwheeling effect; **b** valve opening during systole with full leaflet expansion

High-speed camera recordings were used to compare with the numerical results. The FE model reproduced a realistic and coherent dynamic behavior of the leaflets throughout the cardiac cycle. In particular, the model successfully captured the so-called pinwheeling effect during valve closure–an effect previously documented in the literature (Hatoum et al. 2021), associated with asymmetric leaflet motion and rotation-like deformation patterns in the coaptation region (Fig. 10a).

This feature is particularly relevant since most finite element models reported in the literature tend to produce symmetric leaflet motion during opening and closing, maintaining 120$^\circ$ symmetry throughout the cycle (Li and Sun 2010; Kim et al. 2008; Martin and Sun 2015; Travaglino et al. 2020; Kusner et al. 2021). These models fail to capture the subtle, out-of-phase movements that are typically observed in in vitro settings. In the proposed model, the asymmetric motion of the leaflets arises naturally from the structural and geometric interactions, without imposing artificial constraints. Additionally, during systolic opening, the leaflets fully stretch and flatten against the aortic wall, closely matching experimental observations (Fig. 10b). In contrast, other FE models in the literature show leaflets that remain slightly curved during opening, maintaining a belly-like shape rather than fully unfolding (Kim et al. 2008). The agreement between the virtual and experimental observations for the E=1 configuration supports the ability of the developed FE model to accurately capture the two most relevant valve configurations, valve closure and opening.

#### Leaflet dynamics in elliptical geometry (E=1.5)

The next validation step involved an aortic root with an elliptical annulus geometry corresponding to a large value of E=1.5, for which in-vitro tests were also available using the Heart Valve Pulse Duplicator System. As in the FE model, a rigid elliptical constraint was used to achieve the defined shape.

**Fig. 11:**
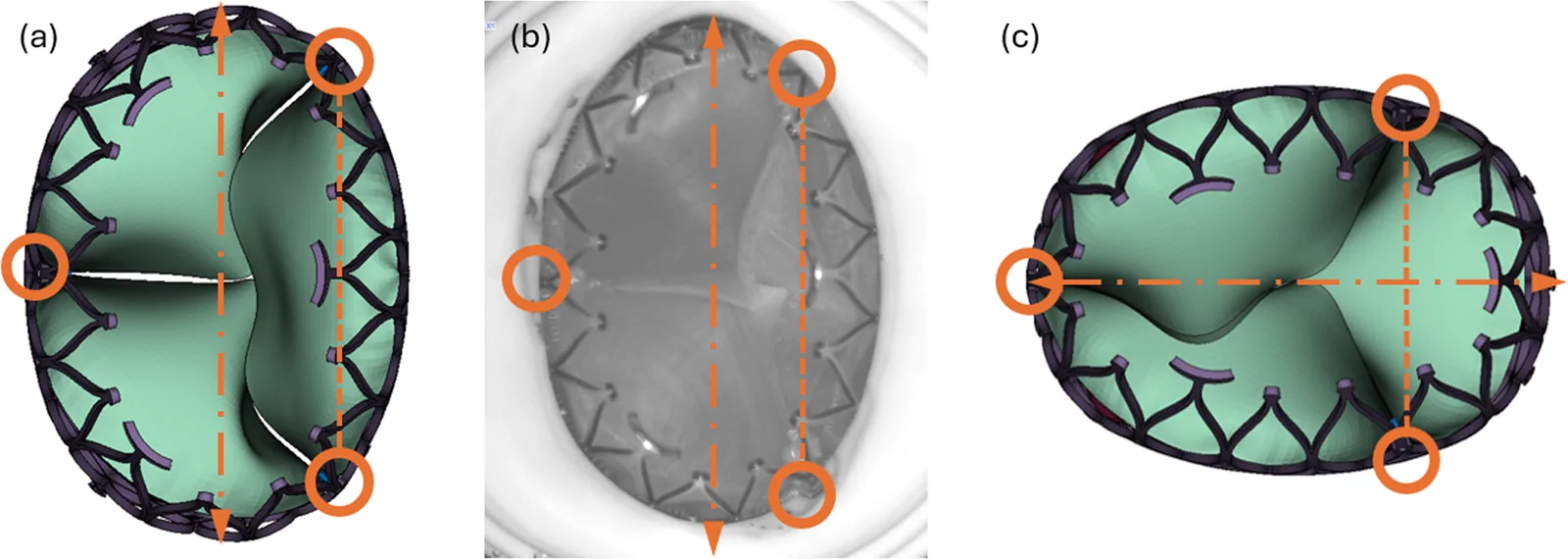
Commissural alignment for the elliptical annulus (E=1.5). **a** FE model with major axis aligned with leaflet commissures (90$^\circ$ orientation); **b** In vitro setup with same alignment; **c** FE model with major axis perpendicular to commissures (0$^\circ$ orientation)

To replicate the experimental setup, the valve was positioned such that the major axis of the elliptical annulus was aligned with the commissural axis of one of the leaflets (Fig. 11a,b) (90$^\circ$ orientation). The FE simulation was carried out for a full cardiac cycle and successfully reproduced key features observed during the in vitro tests. In particular, it captured both the pinwheeling effect during valve closure and the asymmetric opening and closure dynamics of the leaflets.

**Fig. 12:**
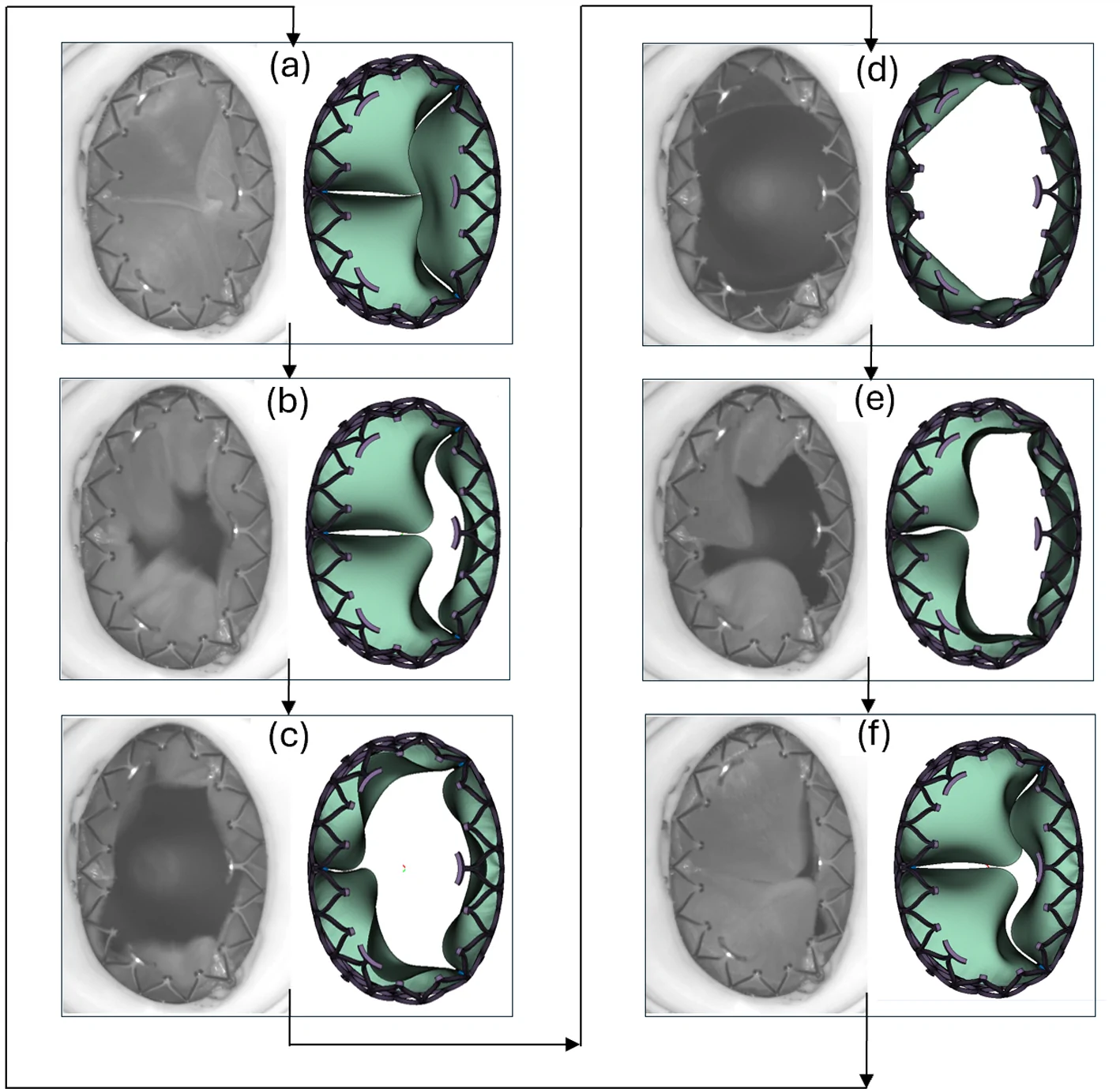
Comparison between the FE model and the in vitro test for E=1.5 (90º orientation): **a**–**d** leaflet opening during systole; **d**–**f** leaflet closure during diastole

Figure 12 presents a side-by-side comparison of axial views from the FE simulation and high-speed camera recordings. Panel (12a) shows the fully closed configuration during diastole, while panel (12d) corresponds to the fully open configuration during systole. Notably, the leaflet whose commissures are aligned with the major axis of the ellipse consistently opens first during systole (12a–12b–12c–12d) and is the last to close during diastole (12d–12e–12f–12a). This early-opening and delayed-closure behavior is clearly observed in the FE and in vitro model, reinforcing the ability of the FE model to reproduce physiologically realistic leaflet motion under non-circular annular conditions.

To further investigate the influence of commissural alignment on leaflet dynamics, an additional FE model was constructed in which the major axis of the elliptical annulus was perpendicular to the line connecting two leaflet commissures (Fig. 11c) (0$^\circ$ orientation). In this configuration an inverse behavior was observed: the leaflet located along the minor axis–being more curved–exhibited a delayed opening during systole and a delayed closure during diastole, compared to the other leaflets.

**Fig. 13:**
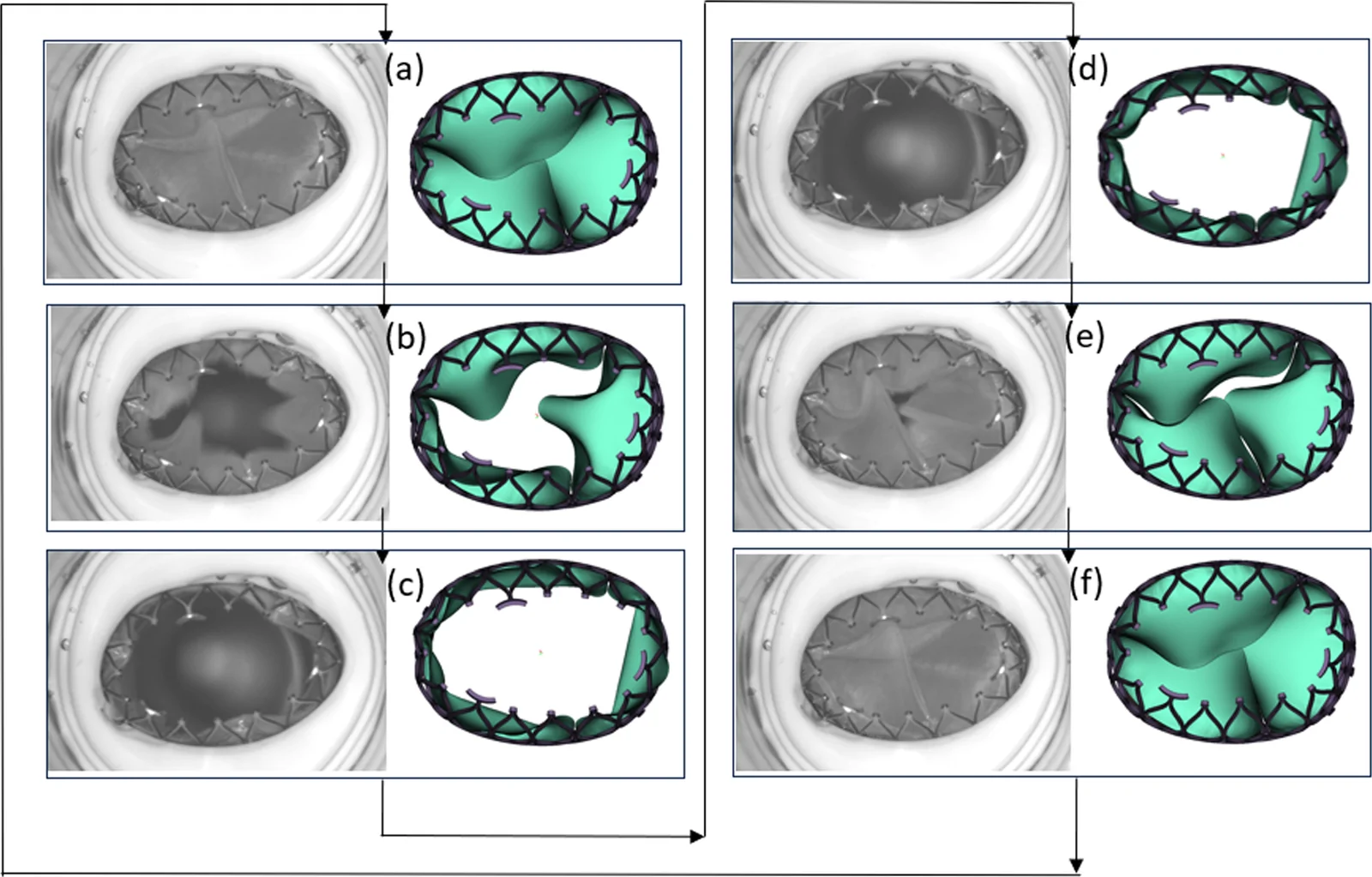
Comparison between the FE model and the in vitro test for E=1.5 (0º orientation): **a**–**d** leaflet opening during systole; **d**–**f** leaflet closure during diastole

This opposite pattern of motion was consistent with observations from the in vitro test performed under the same conditions using the pulse duplicator system (Fig. 13), thereby reinforcing the model’s ability to reproduce realistic dynamic effects induced by annular asymmetry and commissural orientation.

In addition to the qualitative comparison of leaflet motion between the FE model and the in vitro test, a quantitative evaluation of the projected valve open area was also performed. The open area reported in Table 3 was quantified using a custom Python post-processing script developed for this study. For both the experimental recordings and the FE simulation images, the detected open region was normalized with respect to the reference annular area, allowing a direct comparison of the opening ratio during the opening and closing sequence for the $90^\circ$ and $0^\circ$ orientations.

**Table 3:** Comparison of projected valve open area between the in vitro test and FE simulation for the $90^\circ$ and $0^\circ$ orientations

Panel	$\mathbf {90^\circ }$ orientation		$\mathbf {0^\circ }$ orientation	
	TEST %	FEM %	TEST %	FEM %
(a)	0.00	0.00	0.00	0.00
(b)	9.73	9.32	24.33	24.30
(c)	35.47	36.00	56.05	57.24
(d)	61.83	56.82	51.89	50.65
(e)	21.19	32.88	2.07	2.12
(f)	3.00	2.80	0.00	0.00

For both orientations, the FE model reproduced the overall evolution of valve opening and closure observed experimentally, showing a close agreement in the projected open area throughout the cardiac cycle. In the $90^\circ$ orientation, the maximum opening panel (d) was slightly underestimated by the numerical model, while the largest discrepancy was observed during the late closure stage at panel (e). For the $0^\circ$ orientation, the agreement between the experimental and numerical results was particularly close during both opening and closure.

Overall, the quantitative comparison of the projected open area supports the ability of the FE model to reproduce the global leaflet kinematics observed experimentally and to capture the main orientation-dependent effects of annular ellipticity on valve dynamics. Therefore, the present comparison should be interpreted as a semi-quantitative validation of the leaflet kinematics rather than as an exact frame-by-frame reproduction of the experimental motion.

## Results

### Annular geometry effects on valve function

After the finite element model had been compared with the in vitro experiments for both the circular configuration (E = 1) and the highly elliptical configuration (E = 1.5), four additional models were generated with ellipticity values of 1.06, 1.10, 1.20, and 1.35. These additional configurations were used to evaluate the progressive effect of annular ellipticity on valve function. In addition, two different orientations of the elliptical annulus were considered. In the first orientation, the major axis of the ellipse was aligned at 0$^{\circ }$, whereas in the second one, the major axis was rotated by 90$^{\circ }$. This allowed the influence of both the magnitude of ellipticity and its angular orientation with respect to the leaflet arrangement to be assessed.

**Fig. 14:**
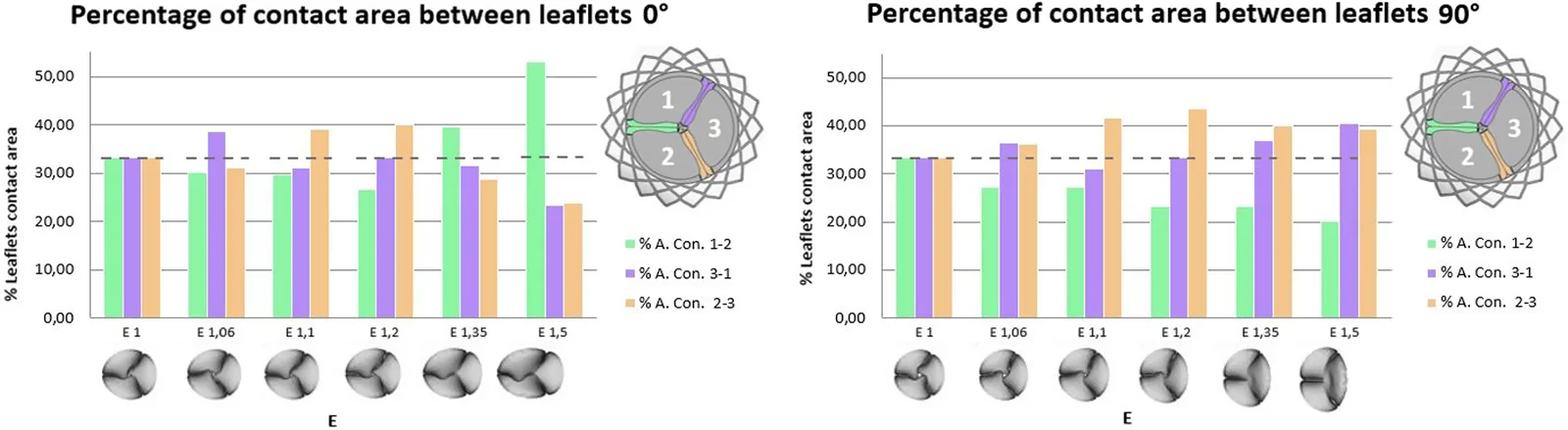
Percentage of contact area between leaflets as a function of ellipticity (*E*)

Leaflet-to-leaflet interaction in the closed configuration was quantified by computing the contact area between each leaflet pair. The percentage of total contact area associated with each leaflet pair was calculated for all ellipticity values and for both annular orientations. The corresponding results are shown in (Fig. 14), together with the final closed configuration of the leaflets for each case.

For the circular annulus, corresponding to E = 1, the contact area was distributed in a nearly balanced manner between the different leaflet pairs. This indicates that, under circular boundary conditions, the three leaflets reached a relatively symmetric closed configuration. As ellipticity increased, however, the contact area distribution became progressively more uneven. This effect was especially evident for the most elliptical configuration, E = 1.5, where the difference in contact area between leaflet pairs became markedly larger.

The orientation of the elliptical annulus also affected the final leaflet configuration. For the same ellipticity value, rotating the major axis of the ellipse by 90$^{\circ }$ changed the way in which the leaflets folded during closure and modified the contact area distribution between leaflet pairs. Therefore, the results show that the final coaptation pattern is not only dependent on the degree of ellipticity, but also on the angular alignment of the elliptical annulus with respect to the valve geometry.

**Fig. 15:**
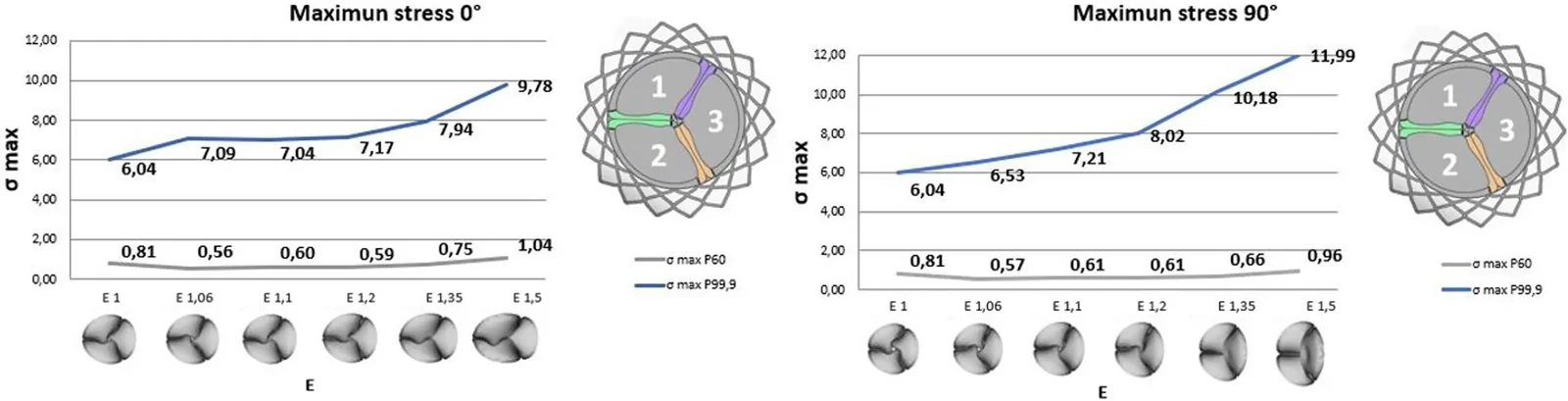
Maximum stresses on the leaflets with P60 and P99.9 for 0$^\circ$ and 90$^\circ$

Leaflet stresses were also evaluated for all ellipticity values and for both orientations. (Fig. 15) shows the P60 and P99.9 stress percentiles for each case. These percentile values were used instead of relying only on the absolute maximum stress in order to reduce the influence of isolated numerical stress peaks and to provide a more representative description of the stress distribution over the leaflet surface.

The leaflet mesh consisted of 18,600 elements. Therefore, the P99.9 value represents a very high stress percentile, with fewer than approximately 20 elements exhibiting stress values above this threshold. In contrast, the P60 value provides information about the stress level below which 60% of the leaflet elements are found, allowing the global shift of the stress distribution to be evaluated.

The results showed a progressive increase in leaflet stress with increasing annular ellipticity. This trend was observed for both the 0$^{\circ }$ and 90$^{\circ }$ orientations. However, the increase in stress was more pronounced when the major axis of the ellipse was rotated by 90$^{\circ }$. This indicates that the 90$^{\circ }$ orientation generated a less favourable mechanical response in terms of leaflet stress, particularly at higher ellipticity values. The P60 values showed that the overall stress distribution shifted towards higher values as ellipticity increased, while the P99.9 values captured the increase in the most highly stressed regions of the leaflets.

## Discussion

The present trend of progressively higher stress and less symmetric closure as ellipticity increases is in line with earlier studies of non-circular valve deployment. Duraiswamy et al. (2016) reported that, among several non-circular annular shapes, the EllipMinor configuration produced the most severe leaflet loading, with a 218% increase in maximum principal stress and an 80% increase in maximum principal strain relative to the circular case. Gunning et al. (2015) similarly showed that eccentric stent distortion produced a “peel-back” coaptation geometry and significantly higher commissural strains than circular deployment under both normotensive and hypertensive conditions. Abbasi and Azadani (2015) further demonstrated that incomplete transcatheter valve expansion alters not only the magnitude but also the location of leaflet loading, with 2–3 mm under-expansion concentrating high stresses at the commissures and 4–5 mm under-expansion shifting them toward the leaflet belly. Jafar et al. (2020) extended this reasoning by showing that under-expansion contributes more strongly to stenosis, whereas non-circularity contributes more strongly to regurgitation, with durability predictors remaining highest in the commissural regions of deformed valves.

These earlier observations are reinforced by more recent TAVR/BAV studies in anatomically challenging deployments. Qiu et al. (2021) showed that elliptical and incomplete stent deployment increases leaflet stress and impairs leaflet kinematics, with the combined penalty more marked in CoreValve than in SAPIEN 3. Oks et al. (2022) found that increasing annular eccentricity lowers geometric orifice area and raises normalized transvalvular pressure gradients, while Pasta et al. (2021) observed slight asymmetric and elliptical expansion of a self-expanding prosthesis in oval bicuspid anatomy, with paravalvular leak occurring in the gap between the aortic wall and the deformed stent frame.

The present study extends that literature in two useful ways. First, the results show that annular ellipticity has a relevant influence not only on leaflet stress, but also on leaflet closure and leaflet-to-leaflet contact. In the circular configuration, the symmetry of the annulus promotes a balanced interaction between the three leaflets, resulting in a more homogeneous distribution of contact area. As the annulus becomes progressively more elliptical, this symmetry is disrupted, leading to an uneven redistribution of contact between leaflet pairs. This imbalance suggests that elliptical annular geometries alter the coaptation mechanism of the valve: in a symmetric circular configuration, the leaflets tend to meet more uniformly during closure, whereas in an elliptical annulus the geometric constraint imposed on leaflet attachment modifies the leaflet folding pattern, so that some leaflet pairs come into contact over a larger area while others show reduced contact. Such behaviour is consistent with previous reports showing altered leaflet kinematics, non-uniform deformation, and impaired valve function under non-circular deployment (Abbasi and Azadani 2015; Duraiswamy et al. 2016; Gunning et al. 2015; Jafar et al. 2020; Qiu et al. 2021; Oks et al. 2022; Sirois et al. 2018), but the present study extends those findings by explicitly quantifying how contact is redistributed among leaflet pairs during closure. In this context, the contact-area data provide a direct structural measure of coaptation imbalance and help explain the relationship between the loss of closure symmetry and the increase in leaflet stress.

Second, the present results show that the biomechanical response depends not only on the magnitude of ellipticity, but also on its orientation. For the same ellipticity value, changing the orientation of the major axis from 0$^{\circ }$ to 90$^{\circ }$ alters the leaflet closure sequence and leads to different closed leaflet configurations and contact area distributions. This indicates that two valves with the same ellipticity may nonetheless produce different leaflet interactions if their major axes are oriented differently. This observation is directionally consistent with Bailey et al. (2017), who found lower leaflet stresses when prosthetic and native leaflets were aligned and higher stresses as rotational alignment deviated, and with Sirois et al. (2018), who explicitly distinguished two ellipse orientations relative to the leaflet coaptation lines and showed that orientation modulates the response of elliptically deployed valves. Jafar et al. (2020) also identified a markedly more adverse non-circular arrangement in which deformation produced particularly unfavourable leaflet mechanics. Although the alignment conventions used in those studies are not identical to the present 0$^{\circ }$/90$^{\circ }$ definition, they support the same central conclusion: ellipticity is not only a magnitude problem, but also an alignment problem. From a biomechanical perspective, this is particularly relevant because annular shape should not be described only by a scalar ellipticity value. The angular orientation of the elliptical deformation must also be considered, since it conditions how the leaflets fold, contact, and transmit loads during closure, which may be especially important in patient-specific geometries where annular deformation is unlikely to be perfectly aligned with an idealized leaflet arrangement.

The stress results further support the idea that increasing ellipticity leads to less favourable mechanical conditions for the leaflets. As the annulus becomes more elliptical, leaflet stress levels increase, indicating that the tissue is subjected to higher mechanical demands. This increase was observed in both the P60 and P99.9 values, suggesting that ellipticity affects not only isolated highly stressed regions, but also the broader stress distribution across the leaflet surface. The stronger stress increase observed for the 90$^{\circ }$ orientation suggests that this configuration induces a more adverse leaflet deformation pattern. Such behaviour is in agreement with previous studies associating eccentric or asymmetric deployment with impaired leaflet kinematics, reduced effective opening, increased gradients, and higher mechanical loading (Pasta et al. 2021; Qiu et al. 2021; Oks et al. 2022; Yeats et al. 2025). Although high stress values do not directly imply tissue failure, sustained or repeated increases in leaflet stress may be relevant for long-term durability.

Overall, these findings highlight the importance of considering both annular ellipticity and its orientation when evaluating valve function. Increasing ellipticity reduces the symmetry of leaflet coaptation, redistributes contact between leaflet pairs, and increases leaflet stresses, particularly when the major axis of the ellipse is oriented unfavourably with respect to the leaflet geometry. This supports the need to include both annular shape and annular alignment in computational assessments of valve performance, especially in cases where non-circular annular deformation may be clinically relevant.

## Limitations

This study has several limitations that should be borne in mind. Firstly, in this study, the aortic constraint was idealized as a cylindrical geometry with a constant elliptical cross-section along the device height. This simplification was adopted to isolate the effect of ellipticity magnitude and orientation on leaflet mechanics and to enable direct comparison with the available in vitro test configurations. However, for a supra-annular valve design, the deployed geometry is not expected to exhibit uniform ellipticity along the entire height of the device. In realistic anatomical conditions, the inflow region may be more strongly constrained by the annulus, whereas the outflow region may remain closer to a circular configuration. Therefore, the use of a uniform elliptical cylinder represents an idealized boundary condition.

Secondly, the simulations employed linear elastic material models. This represents a limitation of the present study, since pericardial tissue exhibits nonlinear and anisotropic behaviour. Nevertheless, this simplification was adopted because the collagen fibre orientation is not controlled or characterized during the valve manufacturing process and this formulation considerably reduces the computational cost of the simulations. Although the adopted material model does not fully reproduce the complete nonlinear behaviour of the tissue, the resulting valve dynamics and leaflet kinematics were verified against in vitro experiments, showing a good agreement with the experimental observations.

Thirdly, the inverse calibration procedure was based on the cantilever bending experiments reported in the literature by (Murdock et al. 2018), and no additional mechanical data from the same tissue samples, such as stress–stretch tensile measurements, were available to further validate the identified parameters.

Finally, we must clarify that, although the finite element model provides useful information on the effect of the annular geometry of the aorta and the orientation of the TAV on valve mechanics, additional experimental or clinical validation would be required to directly relate the predicted stress levels and contact patterns to the long-term performance of the valve, its durability or the risk of dysfunction.

## Conclusion

A finite element model of a 27 mm ALLEGRA device was developed. This required the development of a hybrid shell–membrane finite element model for the pericardium capable of accurately reproducing its bending behavior. The TAV model was validated through in vitro experiments under pulsatile flow conditions. The model successfully reproduced the complex dynamics of the valve leaflets, including the asymmetric “pinwheeling” effect during closure and the full extension of the leaflets during systolic opening. These features were consistent with high-speed camera recordings and were observed for both circular (E=1.0) and elliptical (E=1.5) aortic root geometries.

The orientation of ellipticity is a first-order determinant of leaflet dynamics. This becomes evident when comparing the same ellipticity value in different orientations, both in the FE model and in the real test. For example, at E=1.5, significant differences in leaflet closure are observed. When the major axis of the elliptical annulus is aligned with the commissural axis of one of the leaflets (90$^\circ$ orientation), leaflet 3 is the last to close during diastole, folding over the other two leaflets in the closed position. In contrast, under the 0$^\circ$ orientation, the same leaflet is the first to close, folding underneath the other two in the closed position.

Beyond qualitative validation, the model enabled quantitative analysis of leaflet contact areas during valve closure across a large range of annular ellipticities–well beyond observed clinical practice, but useful as design test cases–(E=1.0 to E=1.5). Although the total contact area between leaflets remained nearly constant, increasing ellipticity led to significant asymmetries in the distribution of contact areas among the three leaflets. For instance, in the most elliptical configuration (E=1.5), one leaflet exhibited more than twice the contact area of another, indicating a potential for uneven mechanical stress and impaired coaptation.

Furthermore, Von Mises stress is strongly affected by both ellipticity and its orientation. Stress increases by more than 63% when ellipticity rises from E=1.0 (circular annulus) to E=1.5. In addition, for the same ellipticity value, the orientation of the ellipse can lead to differences in Von Mises stress exceeding 20% in the most severe orientation.

These results confirm that aortic root geometry plays a critical role in TAV behavior, particularly in the distribution of mechanical loads during valve closure. The developed model provides a robust computational framework for both optimizing valve design and supporting pre-procedural planning in patients with non-circular annuli.

## Data Availability

No datasets were generated or analysed during the current study.

## Appendix A mesh quality parameters for the TAV simulation model

The mesh quality parameters for the Stent part mesh of the TAV are set out in the Table 4.

**Table 4:** Mesh quality parameters for the stent 3D mesh employed

	Aspect ratio	Skewness	Jacobian
Min	1.02	0.00	0.80
Mean	1.18	1.26	0.98
Max	2.21	31.81	1.00

The mesh quality parameters for the leaflets of the TAV are set out in the Table 5.

**Table 5:** Mesh quality parameters for the shell model of the leaflets

	Aspect ratio	Skewness	Jacobian
Min	1.00	0.00	0.91
Mean	1.19	7.17	0.99
Max	2.17	41.36	0.99

The mesh quality parameters for the TAV skirt are set out in the Table 6.

**Table 6:** Mesh quality parameters for 2D skirt mesh employed

	Aspect ratio	Skewness	Jacobian
Min	1.00	0.003	0.70
Mean	1.40	7.85	0.89
Max	3.00	43.55	1.00
